# Using knowledge management tools in the Saudi National Mental Health Survey helpdesk: pre and post study

**DOI:** 10.1186/s13033-019-0288-5

**Published:** 2019-05-10

**Authors:** Maggie Aradati, Lisa Bilal, Mohammad Talal Naseem, Sanaa Hyder, Abdulhameed Al‐Habeeb, Abdullah Al-Subaie, Mona Shahab, Bilal Sohail, Mansoor Baig, Abdulrahman Binmuammar, Yasmin Altwaijri

**Affiliations:** 1 King Salman Center for Disability Research, Riyadh, Saudi Arabia; 2 Biostatistics, Epidemiology and Scientific Computing Department, King Faisal Specialist Hospital and Research Centre, MBC 03, PO Box 3354, Riyadh, 11211 Saudi Arabia; 3 SABIC Psychological Health Research & Applications Chair (SPHRAC), College of Medicine, King Saud University, Riyadh, Saudi Arabia; 4 Mental Health and Social Services Department, Ministry of Health, PO Box 3354, Riyadh, 11211 Saudi Arabia; 5 Edrak Medical Center, PO Box 3354, Riyadh, 11211 Saudi Arabia; 6 Clinical Epidemiology Department, Leiden University Medical Center, Clinical Psychology, Leiden University, PO Box 9500, 2300 Leiden, The Netherlands; 7 Customer Service Department, ADT Security, 615-18th Street S.E, Calgary, Alberta T2E 6J5 Canada

**Keywords:** Public health, Knowledge management, Help desk, Psychiatric epidemiology

## Abstract

**Background:**

With the growth of information technology, there is a need for the evaluation of cost-effective means of monitoring and support of field workers involved in large epidemiological surveys.

**Aim:**

The aim of this research was to measure the performance of a survey help desk that used knowledge management tools to improve its productivity and efficiency. Knowledge management tools are based on information technologies that improve the creation, sharing, and use of different types of knowledge that are critical for effective decision-making.

**Methods:**

The Saudi National Mental Health Survey’s help desk developed and used specific knowledge management tools including a computer file system, feedback from experts and a call ticketing system. Results are based on the analyses of call records recorded by help desk agents in the call ticketing system using descriptive analysis, Wilcoxon rank-sum test (p < 0.01) and Goodman and Kruscal test (gamma). The call records were divided into two phases and included details such as types of calls, priority level and resolution time.

**Results:**

The average time to resolve a reported problem decreased overall, decreased at each priority level and led to increased first contact resolution.

**Conclusion:**

This study is the first of its kind to show how the use of knowledge management tools lead to a more efficient and productive help desk within a health survey environment in Saudi Arabia. Further research on help desk performance, particularly within health survey environments and the Middle Eastern region is needed to support this conclusion.

## Background

The Saudi National Mental Health Survey (SNMHS) conducted a nationally representative psychiatric epidemiological survey of over 4000 participants in Saudi Arabia. This initiative is part of the World Health Organization (WHO) World Mental Health (WMH) Survey Initiative. The SNMHS was launched in 2010 and aimed to (i) estimate the population prevalence of mental health conditions in Saudi Arabia, (ii) model the etiology of individual mental health conditions, (iii) study comorbidity with other psychiatric and health disorders, (iv) estimate the magnitude of disability caused by psychiatric morbidity, and (v) provide health policy makers with service use data and mental health indicators for health decision making and planning. The survey instrument, WMH-Composite International Diagnostic Interview, adapted for Saudi Arabia, was administered face-to-face in respondents’ homes using computer assisted personal interview (CAPI) technology.

Given the magnitude and complexity of our survey, using state of the art technology, a help desk support team was established mainly to facilitate fieldwork operations by recording, tracking and solving reported incidents, such as hardware, software, and logistical issues, encountered by the field staff during the data collection process as well as receiving inquiries from the public.

Help desks are composed of help desk support staff, referred to here as agents, whose primary objective is to serve as a single point of contact for users to gain access to informational technology (IT)-related advice, information and troubleshooting problems [1] and other assistance, often in real-time. Typically, help desks provide frontline support to clients, employees and interested stakeholders and record each contact into an incident database [2]. In any organization, a help desk’s main goal is to provide high quality service in the shortest amount of time and at the lowest cost. Help desks often implement a number of processes and procedures to improve their efficiency, such as incident management (i.e. the recording, tracking, and solving of reported incidents) [2].

Help desks that serve the organization’s employees have a direct effect on the productivity of the business [3]. For example, in the context of a survey environment, employees experiencing technical difficulties during fieldwork (data collection) will consequently experience a delay or impediment in their ability to complete their primary job function. This will in turn have negative consequences on the organization’s ability to meet its goals. However, access to consistent, quick and effective support by a help desk has a direct effect on the employee’s ability to efficiently conduct and/or resume their work [4].

To resolve a problem, an agent must access different information and knowledge sources. These sources range from the agent’s personal insights, experience, and opinions, to files in a computer file system, internet web searches, communication with colleagues, and access to an IT-enabled repository that captures successful solutions to past problems [5]. Therefore, the more help desk agents rely on acquired and available knowledge to resolve incidents, the quicker and more effective their solutions are, and the more productive are the day-to-day operations of the business.

Our survey team used different tools from the conceptual framework of Knowledge Management as a guiding principle to aid the help desk in managing its knowledge. Knowledge management is a discipline that is used to develop, utilize, deliver, and absorb knowledge inside and outside the organization through an appropriate management process to meet current and future needs [6]. The conceptual framework of Knowledge management thus enables a help desk to create, store, make available, and use knowledge [1]. It has been observed that that the use of IT to facilitate knowledge management in support organizations, improves overall business productivity by improving the speed and quality of solutions to users, establishing consistency, as well as increasing employee and customer satisfaction [7].

The implementation of a knowledge-centric help desk has been examined in a number of studies [8]. An important commonality between these studies is their use of electronic knowledge repositories to facilitate the sharing, creation, storage and use of knowledge to aid the agent in the quick resolution of incidents. For example, a study of an organization’s customer support centre found that incidents that were resolved using a knowledge repository resulted in a 10.76% reduction in the time to resolve a problem, compared to incidents resolved without use of the repository [9]. Overall there is a lack of academic studies that examine help desk processes from the knowledge management perspective [8].

To our knowledge, no previous research study has discussed the role and efficiency of a help desk within a large-scale epidemiological survey. In this paper, we show how the use of knowledge management tools to manage knowledge, increased the productivity of a help desk. This paper contributes to previous literature by showing how knowledge management tools improve the efficiency of a help desk over time through the acquisition and management of organizational and employee knowledge, within a health survey environment.

## Methods

The SNMHS had a field team that consisted of over 100 interviewers, six supervisors and two field managers working across different regions of the Kingdom. The interviewers conducted the interview on a laptop using special sample management software tailored for the survey. A help desk team was established to ensure that the field team specifically, and all the project members were supported by technical experts, who managed all the survey software and hardware tools used in the project and successfully resolved any technical and operational issues.

### Knowledge management tools

**Fig. 1:**
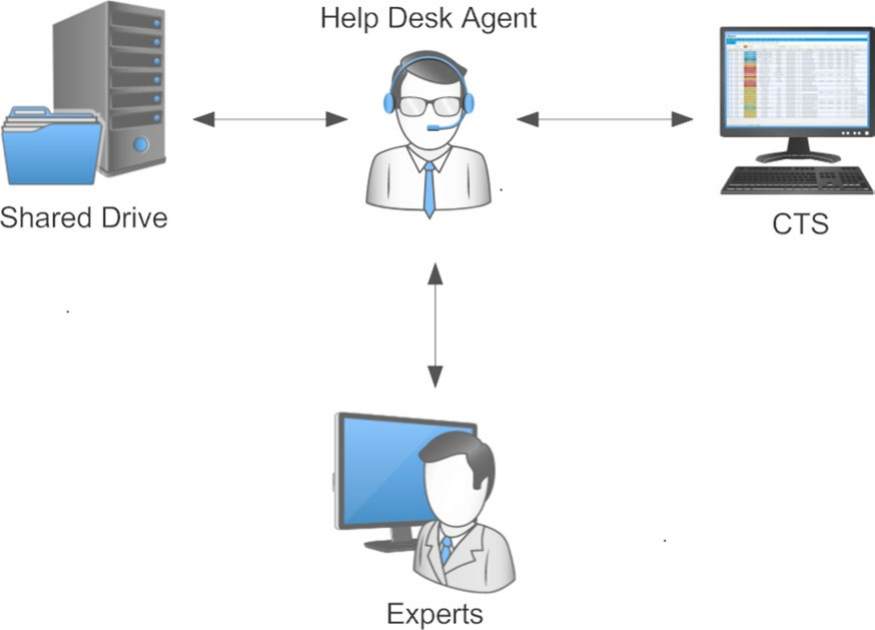
Knowledge management tools used by the SNMHS

Figure 1 depicts the knowledge management tools used by SNMHS, which include (1) a computer file system (shared drive), (2) feedback from experts, and (3) an IT-enabled knowledge repository (Call Ticketing System). The development and use of these tools were based on King Faisal Specialist Hospital & Research Centre’s (Saudi Arabia), internal standard operating procedures. The call ticketing system’s development and implementation was fairly based on Information Technology Infrastructure Library (ITIL) Version 3 best practises [10].

### Computer file system/shared drive

The computer file system consisted of a shared drive which included training and technical manuals, standard operating procedures, and guidebooks related to software use and management, fieldwork procedures, quality control and data management. Examples of the files included are:

- Surveytrak manual: this is a sample management tool used in the survey by the interviewers to record and manage all their interviews and sample; it is also used by the help desk team to replicate any bugs and problems reported by the interviewers.
- Webtrak manual: this is a sample supervision tool used by the help desk agents and fieldwork supervisor to monitor the sample and progress of the interviewers.
- Quality control manual: this contains all the details on quality control checks, procedures and indicators that are used to supervise the interviewers’ performance and work progress.
- Data management: this includes details and instructions for the daily tasks that need to be carried out on all the data related to the interviews and sample; it also includes guidelines for case specific tasks that need to be resolved when needed.
- Training manuals: this contains all the information related to the 2-week training that was given to all the team members and interviewers to understand the survey and fieldwork procedures.
- Standard operating processes: these document all the processes and procedures related to the survey whether technical, administrative, or managerial procedures.

These materials were continuously developed and updated over time (2012–2016, i.e. while fieldwork was actively carried out) by the agents and project team, thus adding to the creation and storage of the knowledge base.

### Call ticketing system (CTS)

Ticketing systems are generally used for capturing and managing incidents; they help provide a knowledge base for the support agent to easily track recorded solutions by searching for similar experiences alongside the users or agents related to the recorded incident [11]. Having these incidents and their troubleshooting steps documented concisely is crucial for efficient work processes, whereby another agent is able to carry out the work by reviewing the ticket [12].

CTS maintains an incident queue and is widely used in product and service sectors, customer/help desk support, and call centers. The main functions are to record, update, and resolve reported issues by the end user. When an incident is reported either via a phone call or email, the agent logs in the event in the problem section and the system automatically generates a unique tracking number so that other agents can easily locate, add to or communicate the status of the reported issue or request. Once the ticket is issued, it is placed in queue under a priority level as high, medium, or low. Critical information is also documented on the CTS because this contains the issues encountered, resolutions, and status, and its assigned support level. A CTS often acts as a knowledge base that contains and archives information on each case or incident with their resolutions in the long run; this enables room for analysing and improving all workflows [11].

The SNMHS strongly believed in the importance of implementing IT best practices; thus, it embedded the CTS into the help desk support process to ensure efficiency when recording, tracking, categorizing and prioritizing issues depending on its severity. The SNMHS ticketing system was a web-based system designed with a friendly user interface, configured and customized according to the scope of the survey and its workflow. Agents were given one-on-one training provided by a help desk support manager to ensure familiarity with the system.

### Experts

The help desk was composed of three expert support levels. The first level included the agents who answer the telephone calls. The second level included senior support and consisted of the help desk data manager, and the survey’s project manager. Finally, the third level comprised of specialists from the University of Michigan in Ann Arbor, USA who did not directly work with the help desk, but were contacted when a technical problem could not be resolved by the in-house team.

### Help desk resolution process flow

**Fig. 2:**
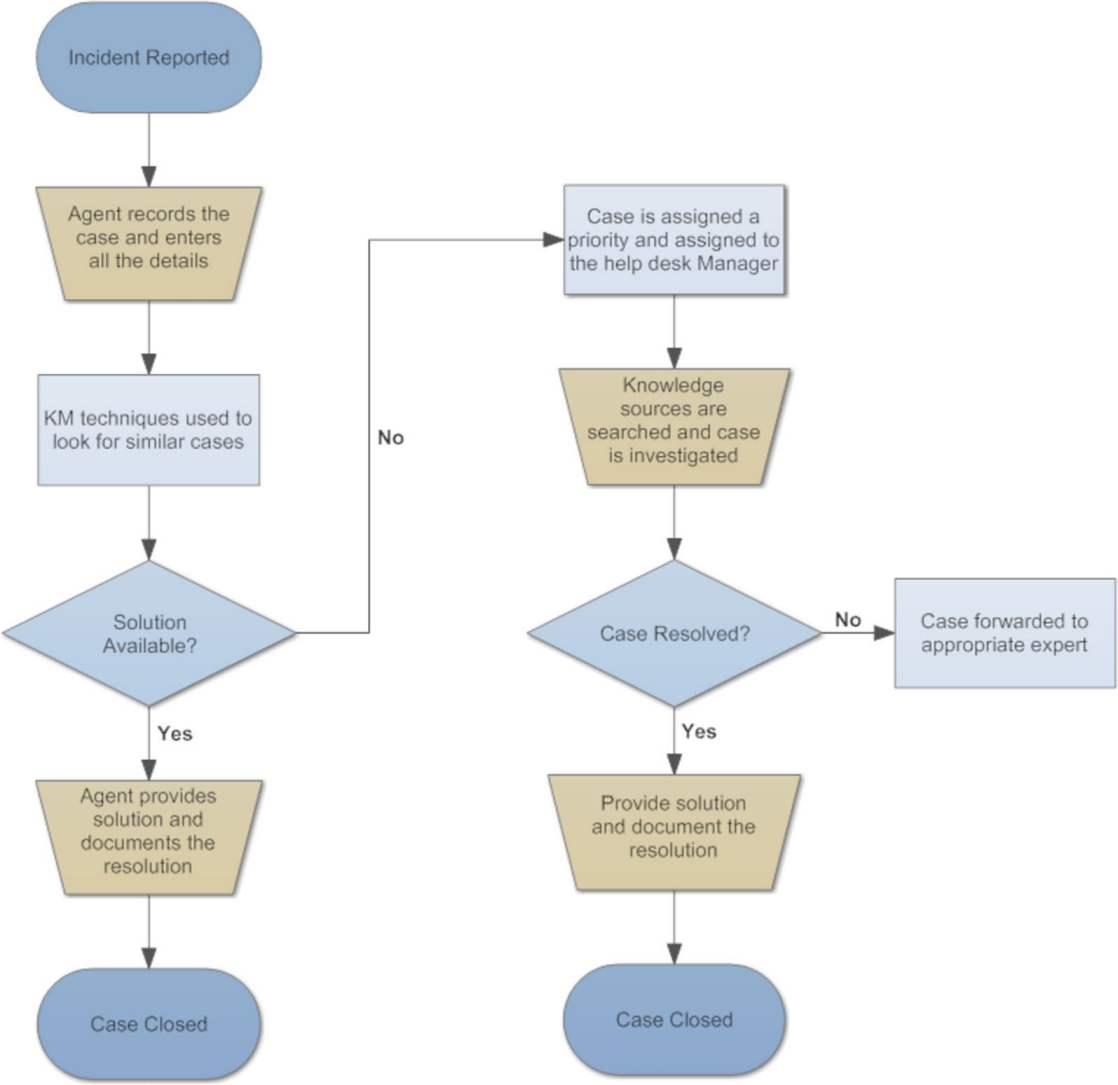
Resolution process flow

The help desk agents followed a specific process for handling the various issues that arose from fieldwork (see Fig. 2). An agent was the first person to receive the call and immediately logged in the basic features of the problem in the CTS. Next, a simple search was performed that shows the history of how similar problems were solved in the past. If no useful results are found to solve the problem, the agent then accessed other knowledge resources, such as the files saved on the computer file system or alternatively decided to escalate the incident to higher levels of support. The process of solving any issue and all the actions taken were recorded in the CTS and closed once the case was finalized.

Similar to other help desks, each case was assigned a priority level according to the following criteria:

#### High priority

A problem that impedes the survey team member’s ability to conduct their work.

#### Medium priority

The problem that does not severely impede the survey team member’s ability to conduct their work and/or can be circumvented.

#### Low priority

A low impact problem that does not require immediate resolution, as it does not directly affect the survey team member’s productivity or software functionality.

As mentioned before, if the agent was unable to find a solution for the problem, then the case was assigned a priority and referred to second level support. The help desk manager or project manager then searched the knowledge resources and investigated the case. If a solution could not be reached, then it was escalated and assigned to the third level. Once a solution was obtained, it was then recorded into the system and the case was closed (see Fig. 2).

### Data collection

The data collected in this paper to measure the efficiency of the Knowledge management tools used by the help desk was extracted from the cases entered in the CTS. These cases covered the data collection period which lasted for 3 years. This period of time was then divided into two phases:

Phase I: from December 2013 to February 2015 which included a total number of 775 cases. Phase II: from March 2015 to December 2016 which included a total number of 216 cases. Phase I marked the period during which the help desk agents had limited knowledge and resources to rely on as the project was in its initial stages and the Knowledge management tools were not thoroughly developed. Whereas in Phase II, the Knowledge management tools described earlier were successfully tested and widely used by the help desk agents.

The key performance indicators to measure help desk performance [2] that were relevant to our study include the number of calls resolved at first contact, and the average time to resolve a problem at each priority level.

The data comprised a total of 991 cases and included details such as the number of resources, types of calls, and resolution time. These cases were then classified into 10 categories as shown in Table 1 with their frequency of occurrence. As shown, most of the cases were related to technical issues as they fall under the Software and Data Management categories, i.e. 26.64% and 22.4% respectively.

**Table 1:** Frequency of cases by category (N = 991)

Category	Frequency	Percent
Software	264	26.64
Data management	222	22.40
Public calls	120	12.11
Fieldwork issues	100	10.09
Logistics	73	7.37
Survey protocol	70	7.06
Hardware	68	6.86
Management	58	5.85
Network connectivity	16	1.61

Table 2 shows the frequency of cases by priority. Most of the cases reported were assigned medium priority and high priority levels, i.e. 55% and 34% respectively.

**Table 2:** Classification of cases by priority (N = 991)

Priority	Frequency	Percent
High	333	34
Low	113	11
Medium	545	55

### Data analysis

All the data collected from the CTS (see Table 3) for the duration of the study was analysed using SAS Enterprise Guide 9.2, a statistical analysis software. SAS Enterprise Guide uses a project based interface to manage data. As the data was divided into two different phases (Phase I and Phase II), it required two different sets of codes, tasks and results. Microsoft Excel 2010 was used to import and export data to and from SAS.

Normality tests were carried on the response times for both phases to determine if they are normally distributed. The tests showed that the data is not normally distributed and a non-parametric test should be used. Wilcoxon rank-sum test was used in SAS enterprise guide 7.1 to generate p-values from the data in both phases. The Wilcoxon rank-sum test was used first to compare all the data from both Phase I and Phase II, and then by priority levels across both phases.

The Goodman and Kruskal’s gamma and the Chi square were used in SAS Enterprise Guide 7.1 to describe the association between the phases and the support levels.

## Results

Table 3 shows the summary output for Phase I and Phase II. The case resolution time referred to the time between logging a problem call and the time the problem is resolved. The case resolution time, reported in hours, is also based on the help desk’s 12-h workday, as opposed to 24 h, and includes downtime. The table shows greater median decrease for the total average case resolution time and at each priority level, except for medium priority cases. These median reductions between Phase I and Phase II are significant for all outputs expect for medium priority cases.

The average time for resolving cases in Phase II was 84.7% lower than Phase I. At the low, medium and high priority level, Phase II outperforms Phase I. Case resolution time for low priority calls was improved by 92.0%, for medium priority by 84.2% and for high priority by 83.8%.

Table 4 shows the percentage of cases that went to each support level. In Phase I, only 38% of the total cases at the support level were presented in Table 4, as this feature was not consistently used by the help desk agents. In Phase II, 100% of the cases at each support level were presented in the table, as this feature was used extensively during this Phase. In Phase II, there was a decrease in the percentages of cases referred to second and third line support, of 66.6% and 92.1% respectively. On the other hand, there was only a 28.9% increase in first level support.

**Table 3:** Summary output for Phase I versus Phase II

Summary output for Phase I versus Phase II	Phase I			Phase II			p-value
	N	Median	IQR	N	Median	IQR	
Total average case resolution time (hours)	755	25	142	214	12.5	25.5	< 0.0001
Total average time for resolving High priority cases (hours)	235	51	159	87	14	43	< 0.0001
Total average time for resolving medium priority cases (hours)	462	21	125	75	5	23	0.1528
Total average time for resolving low priority cases (hours)	58	45	189	52	4.5	19.5	0.0001

Wilcoxon signed-rank test significant at p < 0.01 level

**Table 4:** Percentages of cases referred to each support level

Percentages of cases referred to each support	Phase I		Phase II		p-value	Gamma
	N	%	N	%		
Percentage of cases that went to 3rd level	140	47.3	11	5.1	< 0.0001	
Percentage of cases that went to 2nd level	16	5.4	8	3.7		− 0.8365
Percentage of cases that went to 1st level	140	47.3	197	91.2		

Significant at p < 0.01 level

## Discussion

The aim of the present paper was to show how the use of Knowledge management tools to manage knowledge would increase the productivity of a help desk and would lead to performance improvements. Our findings show that applying knowledge management tools in the duration of active fieldwork decreased the resolution time of high, medium and low priority cases handled by the survey’s help desk.

The results also indicate a large decrease in cases being referred to second and third level support over time and the subsequent increase in cases resolved at first level support. More problems being resolved at the first level could be attributed to the consolidation of knowledge over time and the use of knowledge management tools. This finding is consistent with previous literature [1]. More cases being solved at first level support is an indication of higher productivity, as it translates into more cases being resolved at first contact, and saves time and money, as reflected in the project’s fieldwork operations. With interviewers having to travel to remote places across the country, the quick and effective resolution of incidents reported by the interviewers was crucial for permitting them to conduct and/or resume their work as quickly as possible, saving money on transportation and accommodation costs.

A number of studies have examined the implementation and performance of knowledge management tools in a help desk [13]. The development and implementation of the knowledge management tools and workflows are unique to our study, making direct comparisons difficult. However, when we do compare our findings to other studies that examine a help desk’s efficiency through the use of IT enabled knowledge management tools, we identify a similar impact. In a simulation study of a knowledge management-centric helpdesk, Gonzalez et al. [12] reported improvements at low and high priority levels of 57.9% and 52.2% respectively, which is comparable to the 92.0% and 83.8% found in our study. Spremic et al. [9] analysed the performance of a finance company’s help desk that used a knowledge repository, and reported a 66% decrease in the average time for resolving reported incidents, which is comparable to our findings of an 84.7% decrease. They also report a 19% increase of incidents being resolved at first level support comparable to the 28.9% increase found in our study.

This paper is the first of its kind to measure help desk efficiency and productivity in a health survey research environment in Saudi Arabia. However, it does have some limitations. Because this study is a pre- and post-study, the lack of a control group means that the effects could have occurred naturalistically rather than being caused by the Knowledge management tools. Therefore, it plausible that the improved efficiency observed may have been caused by other variables, such as the agents becoming more familiar with system over time. Another limitation of the study is that 62% of the data in Phase I is not represented in our analysis. Therefore, it is not clear what effect this missing data may have had on the estimated reduction in referrals to higher support levels and the increased first contact resolution. A more complete dataset may have yielded different results in terms of decrease or increase in referrals to support levels.

Furthermore, the findings of the present study may have been enriched if a comparison with a help desk that utilized different knowledge management tools was possible, such as a virtual help desk, e-support systems, or expert systems [1]. Prospective studies should analyse the impact of wide-ranging knowledge management tools on help desk productivity and efficiency, and build upon our findings by creating an efficient help desk using other knowledge management tools.

The present paper does not consider a knowledge management system (KMS) (i.e. a single interface for the help desk agent to access all data, information, and knowledge sources) [12]. Studies have shown that the utilization of KMS improves help desk efficiency by integrating disparate knowledge resources into one system and making the system part of the help desk resolution process to ensure high utilization of the system [12, 14]. KMS also provides access to data on the number of new knowledge materials, how often and when they were utilized, and an option to indicate whether accessed resources were helpful or not. This information would have been used as a success indictor [8] and provided us with a richer analysis, pointing out which types of knowledge management tools were most useful for the resolution process, and which were most important for problem management. Future help desks should consider developing a KMS, especially in the survey context and in the Middle East.

There is a clear need for studies that combine the best practices of knowledge management in a help desk survey setting (e.g. IT infrastructure library-ITIL) [10]. Finally, few studies have examined cultural acceptance of IT among workers in Saudi Arabia [15, 16]. The present paper successfully measures the performance of knowledge management in IT, through a national Saudi survey. However, more research is needed that moves beyond the source to consider the conditions that facilitate knowledge creation [16, 17] especially in the Middle Eastern context.

## Conclusions

This paper is the first of its kind to measure help desk efficiency and productivity in a health survey research environment in Saudi Arabia. The study shows that using a knowledge management tools lead to a greater than 80% decrease in average time to resolve reported incidents and a 28.9% increase in first contact resolution. Based on these significant outcomes, we recommend that others considering to undertake a national household survey, to implement and further develop and test knowledge management practices within their fieldwork operations.

## Data Availability

Please contact corresponding author for data requests.

## Abbreviations

SNMHS: saudi national mental health survey
WHO: World Health Organization
WMH: World Mental Health
CAPI: computer assisted personal interview
CTS: call ticketing system
KMS: knowledge management system

## References

[1] Leung NK, Lau SK (2006). Relieving the overloaded help desk: a knowledge management approach. Commun IIMA..

[2] Office of Government Commerce (2007). ITIL service transition.

[3] Bruton N (2012). How to manage the IT help desk.

[4] Held G (1992). Network management: techniques, tools and systems.

[5] Gray PH, Durcikova A (2005). The role of knowledge repositories in technical support environments: speed versus learning in user performance. J Manag Inf Syst.

[6] Davenport TH, Prusak L (1998). Working knowledge: how organizations manage what they know.

[7] Davenport TH, Klahr P (1998). Managing customer support knowledge. Calif Manag Rev.

[8] Davenport TH, De Long DW, Beers MC (1998). Successful knowledge management projects. Sloan Manag Rev.

[9] Spremic M, Zmirak Z, Kraljevic K. IT and business process performance management: case study of ITIL implementation in finance service industry. In: ITI 2008-30th international conference on information technology interfaces. IEEE; 2008 Jun 23. pp. 243–50.

[10] Adams S. ITIL V3 foundation handbook. London: The Stationery Office; 2009.

[11] Mvungi M, Jay I (2009). Knowledge management model for information technology support service. Electron J Know Manag..

[12] González LM, Giachetti RE, Ramirez G (2005). Knowledge management-centric help desk: specification and performance evaluation. Decis Support Syst.

[13] Jäntti M, Kalliokoski J. Identifying knowledge management challenges in a service desk: A case study. In Information, Process, and Knowledge Management, 2010. eKNOW'10. Second International Conference on 2010 Feb 10 (pp. 100-105). IEEE.

[14] Delic KA, Hoellmer B (2000). Knowledge-based support in help-desk environments. IT Professional..

[15] Al-Gahtani SS, Hubona GS, Wang J (2007). Information technology (IT) in Saudi Arabia: culture and the acceptance and use of IT. Inf Manag.

[16] Hu PJ, Al-Gahtani SS, Hu HF (2014). Arabian workers’ acceptance of computer technology: a model comparison perspective. JGIM.

[17] Alavi M, Leidner DE (2001). Knowledge management and knowledge management systems: conceptual foundations and research issues. MIS Q.

